# The Effects of Conditioned Medium Derived from Mesenchymal Stem Cells Cocultured with Hepatocytes on Damaged Hepatocytes and Acute Liver Failure in Rats

**DOI:** 10.1155/2018/9156560

**Published:** 2018-07-12

**Authors:** Li Chen, Jiexin Zhang, Lu Yang, Guoying Zhang, Yingjie Wang, Shichang Zhang

**Affiliations:** ^1^Department of Obstetrics, The First Affiliated Hospital of Nanjing Medical University, Nanjing 210029, China; ^2^Department of Laboratory Medicine, The First Affiliated Hospital of Nanjing Medical University, Nanjing 210029, China; ^3^Institute of Infectious Diseases, Southwest Hospital, Army Medical University, Chongqing 400038, China

## Abstract

Mesenchymal stem cells (MSCs) and hepatocytes are two attractive sources of cell-based therapies for acute liver failure (ALF). The cotransplantation of hepatocytes with MSCs can improve the therapeutic performance for the treatment of ALF. However, the therapeutic potential of conditioned medium (CM) derived from MSCs cocultured with hepatocytes (MSC-H-CM) remains unclear. The purpose of this study was to investigate the effects of MSC-H-CM on damaged hepatocytes *in vitro* and on D-galactosamine-induced ALF *in vivo*. D-Galactosamine-treated L02 cells cultured in MSC-H-CM exhibited higher of cell viability and total protein synthesis than L02 cells cultured in MSC-CM, CM derived from hepatocytes (H-CM), MSC-CM + H-CM, or with nonconditioned medium (NCM). Lactate dehydrogenase and aspartate aminotransferase levels were lower in the supernatant of damaged L02 cells cultured in MSC-H-CM than in that of L02 cells cultured in other types of CM. The lowest percentage of apoptotic cells was observed after the MSC-H-CM treatment. When CM was injected into the tail vein of rats with ALF, MSC-H-CM was the most successful at preventing the release of liver injury biomarkers and in promoting the recovery of liver structure. The greatest survival rate 7 days after the first treatment was observed in the MSC-H-CM-treated rats. Our results reveal that the delivery of MSC-H-CM could be a novel strategy for integrating the therapeutic potentials of hepatocytes and MSCs for the treatment of ALF.

## 1. Introduction

Liver transplantation is the only long-term effective treatment for acute liver failure (ALF), but it is limited by the shortage of transplantable organs [1]. With the advancement of regenerative medicine, alternatives to liver transplantation are becoming distinct possibilities—be it through hepatocyte transplantation, stem cell therapy, or tissue-engineered grafts [2–4]. The liver is particularly amenable to these cell-based therapies due to its innate capacity for intense regeneration and self-repair [5]. Therefore, cell-based therapies have been proposed as a promising avenue for bridging a patient to either liver transplantation or to native liver recovery through endogenous regeneration [2].

Hepatocytes and mesenchymal stem cells (MSCs) are two attractive sources of cell-based therapies for ALF. Previous studies have demonstrated that hepatocyte transplantation can compensate for liver dysfunction, which plays an important role in cell-based therapies for ALF [6]. However, hepatocyte transplantation is hampered by graft versus host reactions and a shortage of transplantable hepatocytes. In addition, transplanted hepatocytes are unavoidably exposed to the inflammatory conditions of ALF, which could suppress their viability and functions [3]. The transplantation of MSCs derived from different tissues can efficiently rescue ALF through suppressing of liver destruction, supporting resident hepatocyte function, enhancing liver regeneration, and modulating inflammatory and immune reactions by paracrine secretions [7], but MSCs cannot provide timely supportive liver function. Therefore, integrating their therapeutic potentials of hepatocytes and MSCs may be a promising method for the treatment of ALF based on the advantages of timely supportive liver functions, immunosuppression, supported resident hepatocyte functions, and enhanced liver regeneration [8].

Recently, our studies have indicated that hepatocytes cotransplanted with MSCs exhibit superior performance for the treatment of ALF compared with the transplantation of hepatocytes or MSCs alone [9]. MSCs can not only reduce the immune rejection of hepatocytes by the host but also improve viability and function of hepatocytes. Cotransplanted hepatocytes can provide timely support of liver functions. Systemic infusion of conditioned medium (CM) derived from MSCs (MSC-CM) has been shown to exhibit a therapeutic potential similar to that of MSC therapy for the treatment of ALF [10, 11]. However, the effects of CM derived from MSCs cocultured with hepatocytes (MSC-H-CM) on ALF and on resident liver cells have not been explored.

In the present study, we evaluated the effect of MSC-H-CM on D-galactosamine-treated L02 cells *in vitro*. Furthermore, the *in vivo* therapeutic potential of MSC-H-CM was analyzed through administration to rats with D-galactosamine-induced ALF and was compared to that of MSC-CM, CM derived from hepatocytes (H-CM), or MSC-CM + H-CM. Our study attempts to explore the novel strategy of delivering MSC-H-CM, which integrates the therapeutic potentials of hepatocytes and MSCs for the treatment of ALF.

## 2. Materials and Methods

### 2.1. Animals

Male Sprague-Dawley rats weighing 250 to 300 g were used for the ALF experiments. MSCs and hepatocytes were isolated from female Sprague-Dawley rats weighing 60 g to 80 g. Animals were provided by the Laboratory Animal Center of Army Medical University. All procedures followed ethical guidelines and were approved by the Institutional Animal Care and Use Committee of the Army Medical University.

### 2.2. Isolation, Culture, and Coculture of MSCs and Hepatocytes

Bone marrow-derived MSCs were isolated, cultured, and characterized for surface marker expression and adipocytic and osteogenic differentiation capacity as described previously [9]. After 3-4 passages, these cells were used for experiments. Hepatocytes were isolated from Sprague-Dawley rats using a two-step collagenase perfusion procedure as previously described [6]. Six-well plates were used for the coculture system, in which freshly isolated hepatocytes (1 × 10^6^/well) were cocultured with MSCs (0.2 × 10^6^/well) during 3 to 4 passages in Dulbecco's modified Eagle's medium (DMEM; Gibco, Carlsbad, CA, USA) supplemented with antibiotic-antimycotic solution and 10% fetal bovine serum (Gibco) at 37°C in 95% humidified air and 5% CO_2_. Plates containing 1.2 × 10^6^ hepatocytes or MSCs per well were also cultured under identical conditions to serve as controls.

### 2.3. CM Production

For the generation of CM, the abovementioned cells were cultured for 24 hours, washed thoroughly, and cultured in 2 ml of DMEM supplemented with 2% fetal bovine serum and 2 mmol/l L-glutamine (Gibco). The CM was collected 24 hours later and concentrated 25-fold using ultrafiltration units with a 3 kDa cutoff (Millipore, Bedford, MA, USA). The concentrated CM was immediately cryopreserved at −80°C until use. MSC-CM, H-CM, and MSC-H-CM were derived from MSCs, hepatocytes, and a coculture of MSCs and hepatocytes, respectively. The control medium (non-CM or NCM) consisted of a similar medium without conditioning by human MSCs or hepatocytes.

### 2.4. Immunophenotyping by Flow Cytometry Analysis

MSCs were analyzed by flow cytometry, using the following antibodies: CD29-PE, CD45-FITC, and CD34-FITC (all from eBioscience). Adherent BMSCs were detached with 0.25% trypsin (Gibco), washed with phosphate-buffered saline (PBS) three times, centrifuged for 5 minutes at 1200 ×g, and resuspended in PBS. Aliquots containing 5 × 10^5^ cells were incubated for 20 minutes at 4°C with the previously described primary antibodies. The cells were washed and incubated with a corresponding secondary antibody for an additional 20 minutes at 4°C. Finally, the cells were fixed in 10% formalin and analyzed using a cytometer. In each case, 10,000 events were acquired and analyzed by flow cytometry using CellQuest software.

### 2.5. Measurement of Cytokines in CM

Rat Cytokine Antibody Arrays (G series 2; RayBiotech) were used for the qualitative assessment of 34 cytokines in MSC-CM, H-CM, and MSC-H-CM according to the manufacturer's instructions. Briefly, after blocking the array for 30 minutes, 100 *μ*l of CM was incubated overnight at 4°C with an array support labeled with 34 different cytokines. After washing with buffer, a cocktail of biotin-labeled antibody was added, and the array was incubated at room temperature for an additional 1 to 2 hours. The secondary antibody was labeled with streptavidin, and the array was incubated with labeled secondary antibody at room temperature for 1 hour. Signals were detected using the GenePix Array Scanner (Axon Instruments Inc.), and the data were analyzed with a RayBio Analysis Tool and normalized to the average signal intensity of the positive controls for each array. Cytokines in CM were quantified using commercially available ELISA kits (Invitrogen) for interleukin (IL)-6, IL-10, monocyte chemotactic protein-1 (MCP-1), matrix metalloproteinase-8 (MMP-8), tissue inhibitor of metalloproteinase-1 (TIMP-1), tumor necrosis factor-*α* (TNF-*α*), and vascular endothelial growth factor (VEGF) according to the manufacturer's instructions. These experiments were performed with cells from six donors.

### 2.6. D-Galactosamine-Induced L02 Cell Damage Model

L02 cells (Shanghai Institute for Biological Sciences, CAS) were suspended in DMEM supplemented with fetal bovine serum (10%), glutamine (2 mM), penicillin (100 IU/ml), and streptomycin (10 mg/ml) and plated in a 96-well plate at a density of 1 × 10^4^ cells per well. After the establishment of monolayers, the medium was removed and replaced with fresh medium containing 5, 10, 20, or 40 mM D-galactosamine (Sigma, St. Louis, MO, USA). L02 cells were cultured in a 5% CO_2_-humidified incubator at 37°C. Cell morphology was observed with an Olympus phase-contrast microscope. Cell proliferation was detected by the methylthiazolyl-tetrazolium bromide (MTT) method on the following day, and the LD50 of D-galactosamine, which caused cell damage, was measured.

### 2.7. Recovery of Damaged L02 Cells Cultured in Different CMs

L02 cells were divided into two groups: the normal L02 cell group and the damaged L02 cell group. After damaged L02 cells were prepared, they were incubated for 24 hours in fresh DMEM with 20% MSC-CM, H-CM, MSC-H-CM, MSC-CM + H-CM (1 : 5), or NCM (25-fold concentrated). Cell viability was assessed by MTT assay. Cell injury was evaluated by LDH and AST leakage. LDH and AST leakage into the medium was quantified using diagnostic kits for each enzyme. Cell function was assessed using a total protein assay.

### 2.8. Annexin V-FITC Assay

The apoptosis of L02 cells was monitored by FACS analysis using annexin V-propidium iodide staining. Cultured normal or damaged L02 cells were detached, centrifuged, and suspended in PBS and stained with annexin V-FITC and propidium iodide (BD Pharmingen, CA, USA). Apoptotic cells were identified as the annexin V-positive/propidium iodide-negative population. Analyses were performed by the FACSCalibur cytometer (BD, USA) using CellQuest software.

### 2.9. ALF Induction and Treatment

ALF was induced by the intraperitoneal injection of D-galactosamine. We chose a dose of 0.8 g D-galactosamine/kg body weight to induce ALF to achieve an intermediate level of mortality; based on our previous studies [9], this dose ensures that a subgroup of vehicle-treated animals will survive long enough to be analyzed for comparison. After 24 hours, we collected tissue from four animals per group and performed survival analyses with 10 animals per group: (1) H-CM group: injected H-CM; (2) MSC-CM group: injected MSC-CM; (3) MSC-H-CM group: injected MSC-H-CM; (4) MSC-CM + H-CM group: injected MSC-CM + H-CM (1 : 5); and (5) NCM group: injected NCM. Each rat received an injection of 0.8 ml of the corresponding CM three times per day for three consecutive days. During the treatment period, behavioral changes were observed and recorded, and the effect of treatment on survival was recorded. Blood samples were collected at 0 and 72 hours after treatment by tail snip for the analysis of liver enzyme levels. Serum was collected and stored at −20°C for the analysis of AST, ALT, and total bilirubin levels using the Biochemistry Analyzer (Hitachi, Japan). After 7 days of treatment, the entire livers were removed from sacrificed rats and fixed and prepared for hematoxylin-eosin staining.

### 2.10. Statistical Analysis

Statistical analyses were performed using GraphPad Prism 7.01 (GraphPad, San Diego, CA, USA). The data are presented as the mean ± standard deviation. Animal survival was analyzed by log-rank tests, and *P* values are shown. All other data were analyzed by Student's *t*-test, and *P* < 0.05 indicated statistical significance. The Bonferroni correction was used for multiple comparisons.

## 3. Results

### 3.1. Characteristics of Hepatocytes Cocultured with MSCs

**Figure 1:**
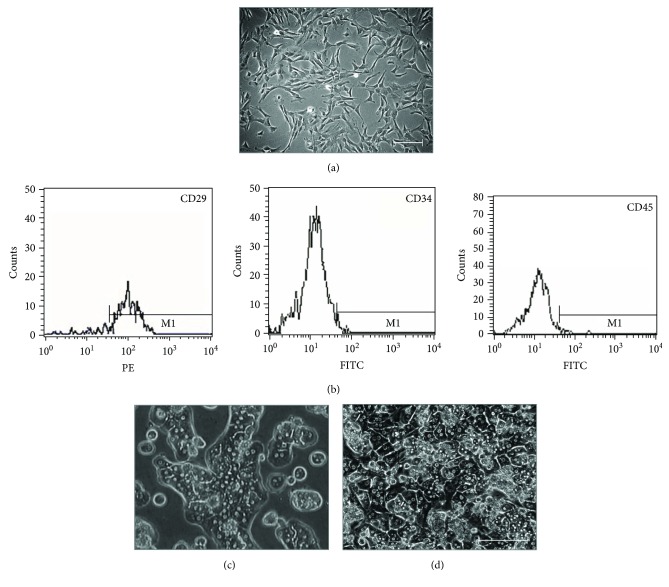
Characterization of isolated MSCs and hepatocytes and cocultured MSCs and hepatocytes. (a) Phase-contrast micrographs of rat MSCs from passage 4 at day 3 of culture. (b) Expression of MSC markers. MSCs were positive for CD29 and negative for CD34 and CD45. (c) Primary hepatocytes exhibited compact and round morphology after 24 hours of monoculture. (d) Hepatocytes cocultured with MSCs displayed a polyhedral shape with well-demarcated cell-cell borders, distinct nuclei, and binucleate. Bar = 100 *μ*m.

Spindle-shaped cells (Figure 1(a)) were positive for the MSC-specific marker CD29, but negative for CD34 and CD45 (Figure 1(b)). *In vitro* differentiation to adipogenic and osteogenic cells was also demonstrated (data not shown). After 24 hours of monoculture, most of the hepatocytes exhibited compact and round morphology, and few cells had an extended shape with apparent nuclei and polyhedral contours (Figure 1(c)). Among hepatocytes cocultured with MSCs, there were a large number of polyhedral cells with well-demarcated cell-cell borders, distinct nuclei, and binucleate, which are typical morphological features of hepatocytes (Figure 1(d)). These observations suggest that cocultured hepatocytes have greater viability than that of monocultured hepatocytes.

### 3.2. Cytokine Profiles of Different CMs

**Figure 2:**
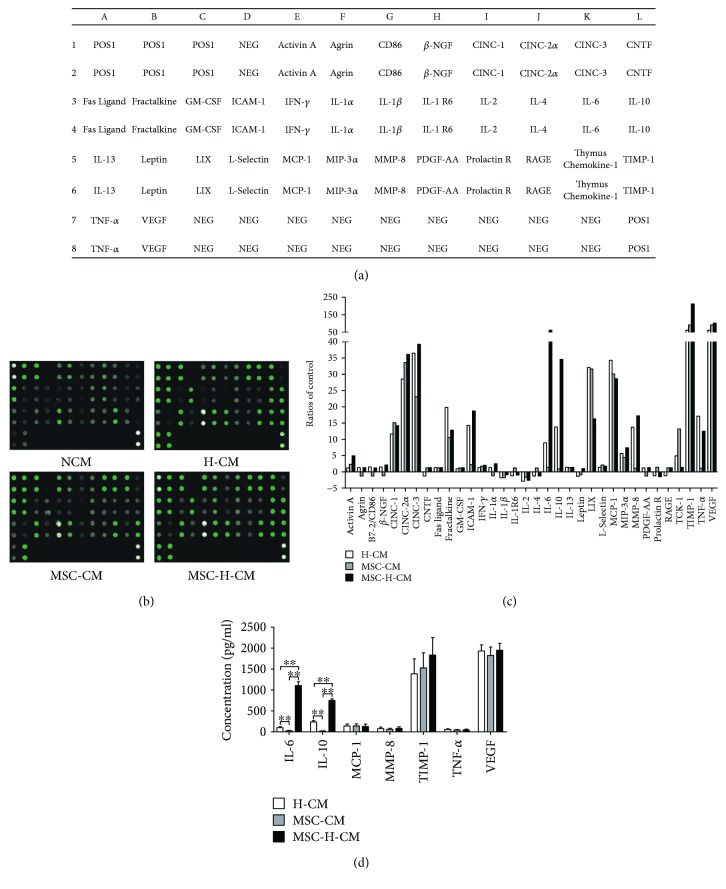
Analysis of cytokines in H-CM, MSC-CM, and MSC-H-CM. (a) Map of 34 cytokines in the rat-specific antibody array. (b) Proteome profile images of cytokines in NCM, H-CM, MSC-CM, and MSC-H-CM. (c) Expression levels of cytokines in H-CM, MSC-CM, and MSC-H-CM relative NCM using a rat-specific antibody array. Protein signals were quantified by the RayBio Analysis Tool and normalized to the average signal intensity of the positive controls for each array. These assays were performed in duplicate. (d) Quantitative results of IL-6, IL-10, MCP-1, MMP-8, TIMP-1, TNF-*α*, and VEGF levels in H-CM, MSC-CM, and MSC-H-CM using ELISA (*n* = 6, ^∗∗^*P* < 0.01).

Studies have demonstrated that MSC-CM can reverse ALF in mice. Cytokine profiles play a key role in the therapeutic effects of different types of CMs. A rat-specific antibody array was used to examine the expression of cytokines in H-CM, MSC-CM, MSC-H-CM, and NCM (Figure 2(a)). The cytokine antibody array showed that H-CM, MSC-CM, and MSC-H-CM have abundant levels of cytokines such as VEGF, TIMP-1, MCP-1, LIX (CXCL5), cytokine-induced neutrophil chemoattractant-1 (CINC-1), CINC-2*α*, and CINC-3 (Figure 2(b)). There were obvious differences in the IL-6 and IL-10 protein levels among the types of CM (Figure 2(c)). The ELISA results revealed that MSC-H-CM had 11.3-fold and 48.1-fold increases in IL-6 levels, and 3.2-fold and 39.4-fold increases in IL-10 levels compared to H-CM and MSC-CM, respectively (Figure 2(d)).

### 3.3. MSC-H-CM Promotes the Recovery of Damaged L02 Cells *In Vitro*

**Figure 3:**
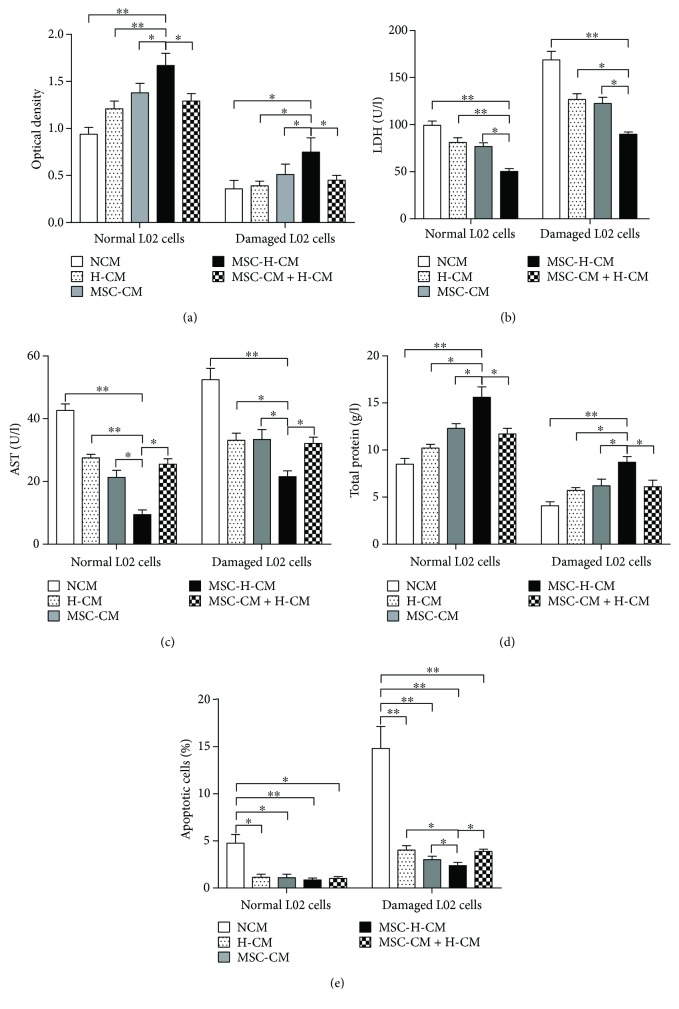
The effects of different CM treatments on damaged hepatocytes *in vitro*. (a) The viability of normal and damaged L02 cells cultured in each CM was examined by MTT assay (*n* = 6, ^∗^*P* < 0.05, and ^∗∗^*P* < 0.01). (b) LDH level in L02 cells cultured in each types of CM. (c) AST level in L02 cells cultured in each types of CM (*n* = 6, ^∗^*P* < 0.05, and ^∗∗^*P* < 0.01). (d) Total protein secreted by L02 cells cultured in each types of CM (*n* = 6, ^∗^*P* < 0.05, and ^∗∗^*P* < 0.01). (e) Comparison of the number of apoptotic L02 cells cultured with different types of CM (*n* = 6, ^∗^*P* < 0.05, and ^∗∗^*P* < 0.01). The apoptosis of L02 cells cultured in different types of CM was determined by FACS analysis using annexin V-propidium iodide staining.

To investigate the effects of each CM on damaged hepatocytes *in vitro*, an MTT assay was used to investigate the viability of L02 cells cultured in different types of CM. Normal L02 cells cultured in MSC-H-CM exhibited higher cell viability than those of L02 cells cultured in MSC-CM, H-CM, MSC-CM + H-CM, or NCM (*P* < 0.05). Significant decreases in cell viability were observed for damaged L02 cells cultured in each type of CM compared with normal L02 cells cultured in the corresponding CM (*P* < 0.05). However, greater cell viability was detected in damaged L02 cells cultured in MSC-H-CM than those cultured in MSC-CM, H-CM, MSC-CM + H-CM, or NCM (*P* < 0.05), suggesting that MSC-H-CM possesses the greatest capacity to improve the viability of injured hepatocytes (Figure 3(a)).

Injured hepatocytes can release LDH and AST into culture medium. LDH and AST levels were higher in the supernatant from damaged L02 cells than in that from normal L02 cells (*P* < 0.01). LDH and AST levels in the supernatant from damaged L02 cells cultured in MSC-H-CM were lower than those in the supernatant from damaged L02 cells cultured with other types of CM (*P* < 0.05) (Figures 3(b) and 3(c)). In addition, total protein synthesis was higher in damaged or normal L02 cells cultured in MSC-H-CM than in corresponding cells cultured in MSC-CM, H-CM, MSC-CM + H-CM, or NCM (*P* < 0.05) (Figure 3(d)). These results indicate that MSC-H-CM is the most effective at enhancing the recovery of damaged hepatocytes.

### 3.4. MSC-H-CM Inhibits the Apoptosis of Damaged L02 Cells *In Vitro*

To evaluate the effect of CM on apoptosis, L02 cells were stained with annexin V-FITC and propidium iodide. Flow cytometric analysis showed that the percentage of apoptotic or necrotic cells was lowest for L02 cells cultured in MSC-H-CM among those cultured in each types of CM. The number of apoptotic L02 cells after culture in MSC-H-CM, MSC-CM, MSC-CM + H-CM, or H-CM was significantly decreased compared with that after culture in NCM (*P* < 0.05). The percentage of apoptotic damaged L02 cells cultured in MSC-H-CM was lower than that of damaged L02 cells cultured in H-CM, MSC-CM, or MSC-CM + H-CM (*P* < 0.05). The lowest number of apoptotic cells was observed in MSC-H-CM, demonstrating the strongest inhibitory effect of MSC-H-CM on the apoptosis of damaged hepatocytes *in vitro* (Figure 3(e)).

### 3.5. MSC-H-CM Administration Reverses ALF

**Figure 4:**
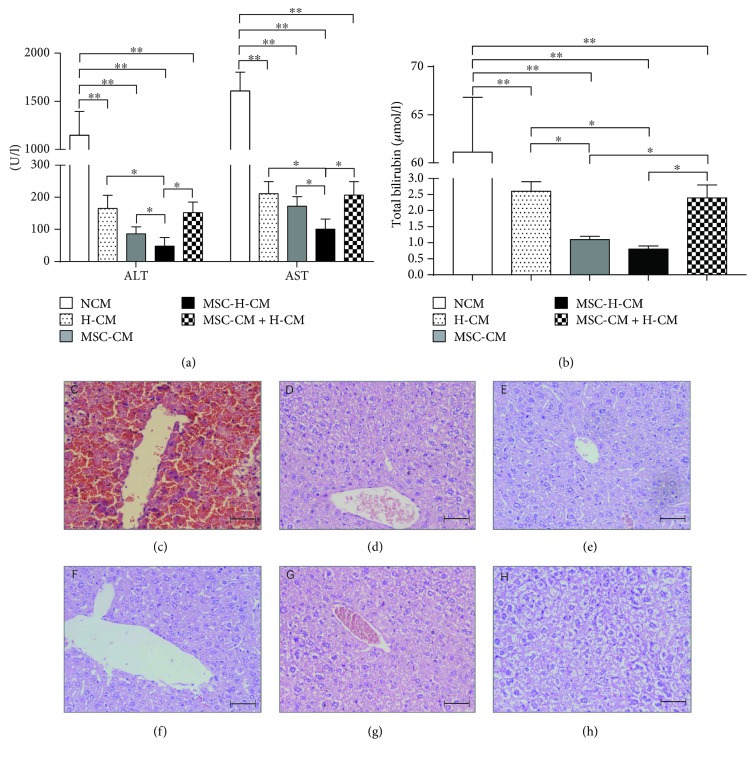
Therapeutic potential of MSC-H-CM for D-galactosamine-induced ALF in rats. (a) and (b) AST and ALT levels (a) release levels and total bilirubin (b) in peripheral blood samples collected 72 hours after the first administration of each type of CM (*n* = 4, ^∗^*P* < 0.05, and ^∗∗^*P* < 0.01). (c–g) Histopathological recovery from D-galactosamine-induced ALF after treatment with each CM. Hematoxylin and eosin staining revealed massive necrosis and hepatic lobule damage in the rat liver after 24 hours of D-galactosamine administration (c). Cell necrosis was completely suppressed in the livers of rat treated with H-CM (d), MSC-CM (e), MSC-H-CM (f), and MSC-CM + H-CM (g) at 7 days after CM injection, compared with those treated with NCM (h). Bar = 50 *μ*m.

Liver enzyme levels in the peripheral blood provide a good estimate of ongoing liver damage. Animal serum was collected at 72 hours after the first CM treatment. As shown in Figure 4(a), serum ALT and AST levels in D-galactosamine-induced ALF rats were increased to 1100 and 1600 IU, respectively, at 72 hours after the administration of NCM, while ALT and AST levels in the rats treated with other types of CM were reduced to below 300 IU. Significant decreases in ALT and AST levels were observed in rats treated with H-CM, MSC-CM, MSC-H-CM, or MSC-CM + H-CM compared with NCM-treated rats (*P* < 0.01). The lowest ALT and AST levels among rats treated with different types of CM were detected in MSC-H-CM-treated rats (*P* < 0.05) (Figure 4(a)). The concentration of total bilirubin was lower in rats treated with MSC-H-CM or MSC-CM than in those treated with H-CM, MSC-CM + H-CM, or NCM (*P* < 0.05). No differences in total bilirubin levels were observed between MSC-H-CM-treated rats and MSC-CM-treated rats (Figure 4(b)). These data demonstrate that the hepatoprotective effect of MSC-H-CM in ALF is greater than that of the other types of CM.

Histological analysis of liver sections revealed massive necrosis and hepatic lobule damage in rat livers at 24 hours after D-galactosamine administration (Figure 4(c)). Cell necrosis was suppressed in the livers of rats treated with H-CM (Figure 4(d)), MSC-CM (Figure 4(e)), MSC-H-CM (Figure 4(f)), or MSC-CM + H-CM (Figure 4(g)) at 7 days after CM injection. By contrast, hepatocellular death with cytoplasmic vacuolization and severe distortion of tissue architecture was observed in NCM-treated rat livers (Figure 4(h)). Furthermore, the liver structure of MSC-H-CM-treated rats recovered from abnormality at 7 days after treatment.

**Figure 5:**
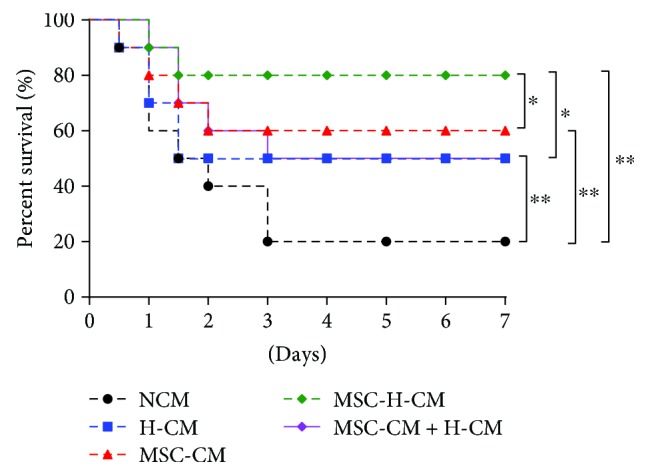
MSC-H-CM increases survival of animals with ALF. Kaplan-Meier survival analysis of rats with D-galactosamine-induced ALF for 7 days after the first treatment of CM (*n* = 10, ^∗^*P* < 0.05, and ^∗∗^*P* < 0.01).

At 7 days after treatment, 80% of the rats treated with MSC-H-CM recovered from ALF, while 50.0%, 50.0%, and 60% of the rats treated with H-CM, MSC-CM + H-CM, or MSC-CM, respectively, recovered from ALF (Figure 5). A significant survival benefit was observed for the H-CM, MSC-CM, MSC-H-CM, and MSC-CM + H-CM groups when compared to the NCM group (*P* < 0.01). The survival rate of the MSC-H-CM group was higher than that of both the H-CM, MSC-CM + H-CM, and MSC-CM groups (*P* < 0.05). There was no significant difference in the survival rate between the H-CM, MSC-CM + H-CM, and MSC-CM groups. Collectively, these data demonstrate that MSC-H-CM is an optimal CM to reverse ALF that exerts a survival benefit.

## 4. Discussion

Cell-based therapies have been proposed as a tangible alternative to liver transplantation for ALF because they are the simpler and less invasive procedures [12]. Paracrine factors derived from MSCs are primarily responsible for the beneficial effects of cell-based therapies in the treatment of ALF [13, 14]. Our previous study demonstrated that MSCs efficiently rescue ALF through paracrine effects rather than hepatic differentiation [6]. Further study showed that the transplantation of cocultured MSCs and hepatocytes provides better restoration of liver function and comparatively less hyperacute rejection in ALF mice [9]. MSC-CM, including their secreted factors, microvesicles and exosomes, exhibits an effect similar to that of MSCs for the treatment of ALF [11, 15–18]. The administration of CM can overcome the genomic instability, immune reactivity, and tumorigenic potential of stem cell transplantation [14]. Thus, the therapeutic potential of CM derived from different cells is attracting increased attention in the field of cell-based therapy [19, 20]. However, the therapeutic potential of MSC-H-CM has not been reported for ALF. In this study, we demonstrated that MSC-H-CM not only promoted the recovery of damaged hepatocytes *in vitro* but also exhibited superior performance in rescuing D-galactosamine-induced ALF, suggesting that MSC-H-CM can be used as a novel tool for integrating the therapeutic potentials of hepatocytes and MSCs for the treatment of ALF.

ALF is typically associated with numerous damaged hepatocytes and massive hepatocellular necrosis [21]. Consequently, the recovery of damaged hepatocytes and the inhibition of cell death should play an important role in cell-based therapies for ALF [22]. Our results indicate that among MSC-CM, H-CM, MSC-CM + H-CM, MSC-H-CM, and NCM treatments, MSC-H-CM has the greatest capacity to enhance the recovery of damaged L02 cells and inhibit the apoptosis of damaged L02 cells *in vitro*. The observed beneficial effects of MSC-H-CM on damaged hepatocytes indicate that it is a potential treatment for ALF.

In our *in vivo* study, the levels of AST, ALT, and total bilirubin, three important indicators of liver injury, were significantly decreased after the administration of MSC-H-CM, MSC-CM, MSC-CM + H-CM, or H-CM. The magnitude of the improved liver functions was higher in MSC-H-CM-treated rats than in rats treated with other types of CM, indicating that the therapeutic potential of CM as a cell-based therapy of ALF was enhanced by coculturing MSCs with hepatocytes. Histological evaluation of liver tissue after CM treatment provided initial insights into the cellular target. A striking recovery of liver structure was seen after MSC-H-CM injection, suggesting that MSC-H-CM may facilitate endogenous liver regeneration. These results are consistent with our *in vitro* data that MSC-H-CM can inhibit the necrosis of damaged hepatocytes and can promote endogenous liver regeneration. Furthermore, MSC-H-CM showed more potential than MSC-CM, H-CM, or MSC-CM + H-CM to reverse liver failure in a rat model of D-galactosamine-induced ALF. The therapeutic potential of CM can be improved by coculturing MSCs with hepatocytes.

The therapeutic benefit of CM is attributed to the paracrine effects of cells in the cell-based therapy of ALF [23]. Different components of CM from different cell types may have different effects in the treatment of ALF [19, 20, 24]. We found high protein levels of VEGF, TIMP-1, MCP-1, LIX, CINC-1, CINC-2*α*, and CINC-3 in all of the studied types of CM. Previous studies have demonstrated that the ability of MSCs or MSC-CM to treat ALF can be attributed to several cytokines, including VEGF, TIMP-1, and MCP-1 [17, 25–27]. However, IL-6 and IL-10 protein levels were significantly increased in MSC-H-CM compared with H-CM or MSC-CM. Studies have indicated that IL-6 and IL-10 secreted from MSCs are responsible for liver recovery in mice with ALF [23, 28]. Studies blocking IL-10 or IL-6 secretion from these cells confirmed the therapeutic potential of the indicated cytokines in mouse ALF models [23]. Therefore, the differences in these cytokines in MSC-H-CM may result in a greater therapeutic effect against ALF than observed with H-CM or MSC-CM, suggesting that the coculture of MSCs with hepatocytes can improve the capacity of CM to reverse ALF.

In summary, the present study shows that MSC-H-CM, which integrates the therapeutic potentials of hepatocytes and MSCs, has superior performance at promoting the recovery of damaged hepatocytes *in vitro* and reversing D-galactosamine-induced ALF *in vivo*. This work provides the first evidence that the delivery of MSC-H-CM may be a novel strategy for integrating the therapeutic potentials of hepatocytes and MSCs for the treatment of ALF, thereby avoiding the shortcomings of cell transplantation.

## Data Availability

The data used to support the findings of this study are available from the corresponding author upon request.

## References

[1] Zern M. A. (2009). Cell transplantation to replace whole liver transplantation. *Gastroenterology*.

[2] Nicolas C. T., Hickey R. D., Chen H. S. (2017). Concise review: liver regenerative medicine: from hepatocyte transplantation to bioartificial livers and bioengineered grafts. *Stem Cells*.

[3] Dhawan A., Puppi J., Hughes R. D., Mitry R. R. (2010). Human hepatocyte transplantation: current experience and future challenges. *Nature Reviews Gastroenterology & Hepatology*.

[4] Trounson A., McDonald C. (2015). Stem cell therapies in clinical trials: progress and challenges. *Cell Stem Cell*.

[5] Singhal A., Neuberger J. (2007). Acute liver failure: bridging to transplant or recovery– are we there yet?. *Journal of Hepatology*.

[6] Zhang S., Chen L., Liu T. (2012). Human umbilical cord matrix stem cells efficiently rescue acute liver failure through paracrine effects rather than hepatic differentiation. *Tissue Engineering Part A*.

[7] Volarevic V., Nurkovic J., Arsenijevic N., Stojkovic M. (2014). Concise review: therapeutic potential of mesenchymal stem cells for the treatment of acute liver failure and cirrhosis. *Stem Cells*.

[8] Yagi H., Parekkadan B., Suganuma K. (2009). Long-term superior performance of a stem cell/hepatocyte device for the treatment of acute liver failure. *Tissue Engineering Part A*.

[9] Liu M., Yang J., Hu W., Zhang S., Wang Y. (2016). Superior performance of co-cultured mesenchymal stem cells and hepatocytes in poly(lactic acid-glycolic acid) scaffolds for the treatment of acute liver failure. *Biomedical Materials*.

[10] Xagorari A., Siotou E., Yiangou M. (2013). Protective effect of mesenchymal stem cell-conditioned medium on hepatic cell apoptosis after acute liver injury. *International Journal of Clinical and Experimental Pathology*.

[11] Chen G., Jin Y., Shi X. (2015). Adipose-derived stem cell-based treatment for acute liver failure. *Stem Cell Research & Therapy*.

[12] Forbes S. J., Gupta S., Dhawan A. (2015). Cell therapy for liver disease: from liver transplantation to cell factory. *Journal of Hepatology*.

[13] Shi D., Zhang J., Zhou Q. (2017). Quantitative evaluation of human bone mesenchymal stem cells rescuing fulminant hepatic failure in pigs. *Gut*.

[14] Gazdic M., Arsenijevic A., Markovic B. S. (2017). Mesenchymal stem cell-dependent modulation of liver diseases. *International Journal of Biological Sciences*.

[15] Chen L., Xiang B., Wang X., Xiang C. (2017). Exosomes derived from human menstrual blood-derived stem cells alleviate fulminant hepatic failure. *Stem Cell Research & Therapy*.

[16] Cha J. M., Shin E. K., Sung J. H. (2018). Efficient scalable production of therapeutic microvesicles derived from human mesenchymal stem cells. *Scientific Reports*.

[17] Lotfinia M., Kadivar M., Piryaei A. (2016). Effect of secreted molecules of human embryonic stem cell-derived mesenchymal stem cells on acute hepatic failure model. *Stem Cells and Development*.

[18] Phinney DG, Pittenger MF (2017). Concise Review: MSC-Derived Exosomes for Cell-Free Therapy. *Stem Cells*.

[19] Wang S., Lee J. S., Hyun J. (2015). Tumor necrosis factor-inducible gene 6 promotes liver regeneration in mice with acute liver injury. *Stem Cell Research & Therapy*.

[20] Fouraschen S. M. G., Pan Q., de Ruiter P. E. (2012). Secreted factors of human liver-derived mesenchymal stem cells promote liver regeneration early after partial hepatectomy. *Stem Cells and Development*.

[21] Bernal W., Auzinger G., Dhawan A., Wendon J. (2010). Acute liver failure. *The Lancet*.

[22] van Poll D., Parekkadan B., Cho C. H. (2008). Mesenchymal stem cell-derived molecules directly modulate hepatocellular death and regeneration in vitro and in vivo. *Hepatology*.

[23] Zagoura D. S., Roubelakis M. G., Bitsika V. (2012). Therapeutic potential of a distinct population of human amniotic fluid mesenchymal stem cells and their secreted molecules in mice with acute hepatic failure. *Gut*.

[24] Chang W. J., Song L. J., Yi T. (2015). Early activated hepatic stellate cell-derived molecules reverse acute hepatic injury. *World Journal of Gastroenterology*.

[25] Banas A., Teratani T., Yamamoto Y. (2008). IFATS collection: in vivo therapeutic potential of human adipose tissue mesenchymal stem cells after transplantation into mice with liver injury. *Stem Cells*.

[26] Moslem M., Valojerdi M. R., Pournasr B., Muhammadnejad A., Baharvand H. (2013). Therapeutic potential of human induced pluripotent stem cell-derived mesenchymal stem cells in mice with lethal fulminant hepatic failure. *Cell Transplantation*.

[27] Salomone F., Barbagallo I., Puzzo L., Piazza C., Li Volti G. (2013). Efficacy of adipose tissue-mesenchymal stem cell transplantation in rats with acetaminophen liver injury. *Stem Cell Research*.

[28] Wang J., Ren H., Yuan X., Ma H., Shi X., Ding Y. (2018). Interleukin-10 secreted by mesenchymal stem cells attenuates acute liver failure through inhibiting pyroptosis. *Hepatology Research*.

